# The microbial selenoproteome of the Sargasso Sea

**DOI:** 10.1186/gb-2005-6-4-r37

**Published:** 2005-03-29

**Authors:** Yan Zhang, Dmitri E Fomenko, Vadim N Gladyshev

**Affiliations:** 1 Department of Biochemistry, University of Nebraska, Lincoln, NE 68588-0664, USA

## Abstract

**Background:**

Selenocysteine (Sec) is a rare amino acid which occurs in proteins in major domains of life. It is encoded by TGA, which also serves as the signal for termination of translation, precluding identification of selenoprotein genes by available annotation tools. Information on full sets of selenoproteins (selenoproteomes) is essential for understanding the biology of selenium. Herein, we characterized the selenoproteome of the largest microbial sequence dataset, the Sargasso Sea environmental genome project.

**Results:**

We identified 310 selenoprotein genes that clustered into 25 families, including 101 new selenoprotein genes that belonged to 15 families. Most of these proteins were predicted redox proteins containing catalytic selenocysteines. Several bacterial selenoproteins previously thought to be restricted to eukaryotes were detected by analyzing eukaryotic and bacterial SECIS elements, suggesting that eukaryotic and bacterial selenoprotein sets partially overlapped. The Sargasso Sea microbial selenoproteome was rich in selenoproteins and its composition was different from that observed in the combined set of completely sequenced genomes, suggesting that these genomes do not accurately represent the microbial selenoproteome. Most detected selenoproteins occurred sporadically compared to the widespread presence of their cysteine homologs, suggesting that many selenoproteins recently evolved from cysteine-containing homologs.

**Conclusions:**

This study yielded the largest selenoprotein dataset to date, doubled the number of prokaryotic selenoprotein families and provided insights into forces that drive selenocysteine evolution.

## Abstract 2

An analysis of the selenoproteome of the largest microbial sequence dataset, the Sargasso Sea environmental genome sequences, identified 310 selenoprotein genes that clustered into 25 families. This included 101 new selenoprotein genes that belonged to 15 families, doubling the number of prokaryotic selenoprotein families.

## Background

Selenium is a biological trace element with significant health benefits [1]. This micronutrient is incorporated into several proteins in bacteria, archaea and eukaryotes as selenocysteine (Sec), the 21st amino acid in proteins [2,3]. Sec is encoded by a UGA codon in a process that requires translational recoding, as UGA is normally read as a stop codon [4]. The Sec UGA codon was the first addition to the universal genetic code since the code was deciphered in the mid-1960s [5]. Recently, an additional amino acid, pyrrolysine (Pyl), has been identified, which has expanded the genetic code to 22 amino acids [6,7]. Pyl is inserted in response to a UAG codon in several methanogenic archaea, but the specific mechanism of insertion of this amino acid into protein is not yet known.

The mechanism of selenoprotein synthesis in prokaryotes was elucidated extensively by Böck and colleagues [8,9]. Translation of selenoprotein mRNA requires both a selenocysteine insertion sequence (SECIS) element, which is a *cis*-acting stem-loop structure residing within selenoprotein mRNAs [4,10], and *trans*-acting factors dedicated to Sec incorporation [11]. In eukaryotes and archaea, SECIS elements are located in 3'-untranslated regions (3' UTRs) [12]. Bacterial SECIS elements differ from those in eukaryotes and archaea in terms of sequence and structure and are located immediately downstream of Sec UGA codons in the coding regions of selenoprotein genes [13,14].

As UGA has the dual function of inserting Sec and terminating translation, and only the latter function is recognized by available annotation programs, selenoprotein genes are almost universally misannotated in sequence databases [15]. To address this problem, various computational approaches to predict selenoprotein genes have been developed [16-21]. These programs successfully identified new selenoproteins in mammalian and *Drosophila *genomes and in several EST databases. However, due to lack of bacterial consensus SECIS models, prediction of bacterial selenoproteins in genomic sequences is difficult. Instead, these proteins can be identified through searches for Sec/Cys pairs in homologous sequences [22].

We report here the use of a modified search strategy to characterize the selenoproteome of the largest prokaryotic sequencing project, the 1.045 billion nucleotide whole genome shotgun sequence of the Sargasso Sea microbial populations [23]. This database contains sequences from over 1,800 microbial species, including 148 novel bacterial phylotypes. We detected all known prokaryotic selenoproteins present in this dataset and identified a large number of additional selenoprotein genes. This approach provided a relatively unbiased way to examine the diversity of selenoprotein families and their evolution, and to analyze the composition of the Sargasso Sea microbial selenoproteome as compared with that in the combined set of completely sequenced prokaryotic genomes.

## Results

### Identification of selenoprotein genes in the Sargasso Sea environmental genome database

**Figure 1:**
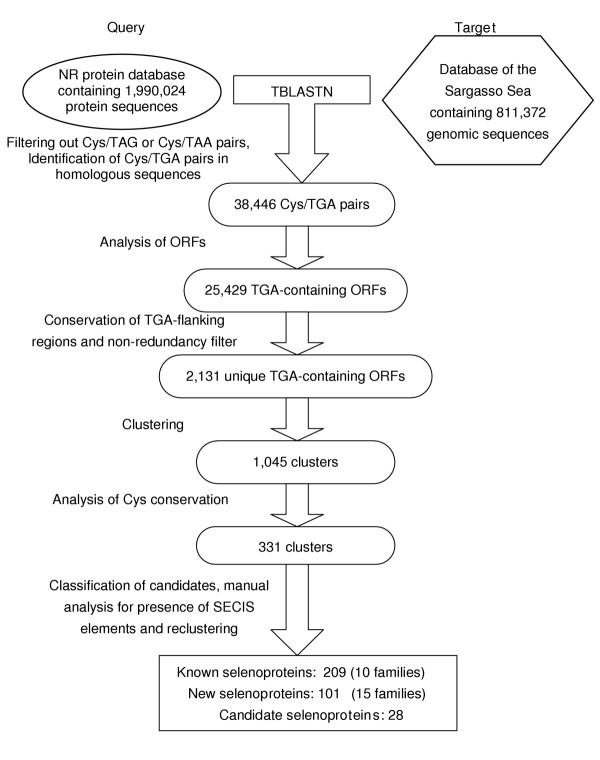
A schematic diagram of the search algorithm. Details of the search process are provided in Materials and methods and are discussed in the text.

The Sargasso Sea genomic database contains the largest collection of microbial sequences derived from a single study [23]. No genes encoding Sec-containing proteins were previously identified and annotated in this dataset. To identify selenoprotein genes in the Sargasso Sea microbial sequences, we used an algorithm that searches for conserved Sec/Cys pairs in homologous sequences. This approach takes advantage of the fact that almost all selenoproteins have homologs (often in different organisms) in which Cys occupies the position of Sec. The methodology is described in Materials and methods and is shown schematically in Figure 1. Briefly, we searched for nucleotide sequences from the Sargasso Sea database which, when translated, aligned with protein sequences from the nonredundant (NR) database such that translated TGA codons aligned with Cys and these pairs were flanked on both sides by conserved sequences. Each TGA-containing sequence in the Sargasso Sea database that was identified in this manner was further screened against a set of filters, which analyzed for possible open reading frames (ORFs), conservation of TGA codons, conservation of Cys in homologs, conservation of TGA-flanking regions in different reading frames and for redundancy. Nonredundant hits were clustered into protein families and a second BLAST search was performed against microbial genomes and NR databases. Finally, all groups of hits were analyzed manually and divided into homologs of previously known selenoproteins, new selenoproteins and selenoprotein candidates.

This procedure identified 209 selenoprotein genes, which belonged to ten known selenoprotein families and 101 selenoprotein genes, which belonged to 15 new selenoprotein families (each represented by at least two sequences) (Table 1). In addition, we detected 28 sequences, which showed homology neither to known and new selenoproteins nor to each other, and these were designated as candidate selenoproteins. Considering that several known selenoproteins were also represented by single sequences (for example, glycine reductase selenoprotein A and glycine reductase selenoprotein B), some of these 28 candidate selenoproteins may be true selenoproteins. However, at present, sequencing errors that generate in-frame TGA codons cannot be excluded and therefore, no definitive conclusions can be made regarding these sequences. Predicted selenoproteins, particularly those represented by a small number of sequences, require future experimental verification.

In total, 310 known and new selenoprotein genes and 28 candidate selenoprotein genes were detected. All these genes were misannotated in the Sargasso Sea dataset, because the previously used annotation tools recognized Sec-encoding TGA codons as terminators. Consequently, some selenoprotein ORFs were annotated as truncated proteins lacking either carboxy-terminal or amino-terminal regions containing Sec, whereas other selenoprotein ORFs were missed altogether.

### Previously known selenoprotein families detected in the Sargasso Sea database

Our procedure detected all known prokaryotic selenoprotein genes present in the Sargasso Sea database, which could also be independently identified by homology searches using known selenoprotein sequences as queries. Eight of the ten known selenoprotein families detected in the dataset were represented by 5-48 selenoprotein genes, whereas two families, glycine reductase selenoprotein A (grdA) and glycine reductase selenoprotein B (grdB), were represented by single sequences. Interestingly, although all known selenoproteins present in the dataset were identified, only nine of the ten families had Cys homologs in the NR database. One selenoprotein, grdA, did not have known Cys homologs [22]. Nevertheless, grdA was also identified because of annotation errors, as Sec in this protein was annotated as Cys in some NR database entries.

Several selenoprotein families had a particularly high representation in the Sargasso Sea dataset. The most abundant family was SelW-like, which contained 48 genes. Although the function of this protein is unclear, a conserved CXXU motif (Cys separated from Sec by two other residues) suggests a redox function. In addition, this protein was previously found to interact with glutathione, a major redox thiol compound in cells [24,25]. A peroxiredoxin (Prx) family had 43 genes and was the second most abundant selenoprotein family. Peroxiredoxins protect bacterial and eukaryotic cells against oxidative injury [26]. Proline reductase (prdB, 42 genes) and selenophosphate synthetase (28 genes) were the third and fourth most abundant families. The former is involved in amino-acid metabolism and catalyzes the reductive ring cleavage of D-proline to 5-aminovalerate [27]. The latter is a key component in prokaryotic selenoprotein biosynthesis [2,28]. A Prx-like protein family was represented by 22 selenoprotein sequences. It had distant homology to the Prx family, but its predicted active site contained a thioredoxin-like UXXC motif instead of the TXXU motif present in Sec-containing Prx. These five families accounted for 87.6% of known selenoprotein sequences, suggesting importance of their functions in the Sargasso Sea environment. Other detected selenoprotein families included thioredoxin (Trx), formate dehydrogenase alpha chain (fdhA), glutathione peroxidase (GPx), grdA and grdB.

### New selenoprotein families identified in the Sargasso Sea database

**Figure 2:**
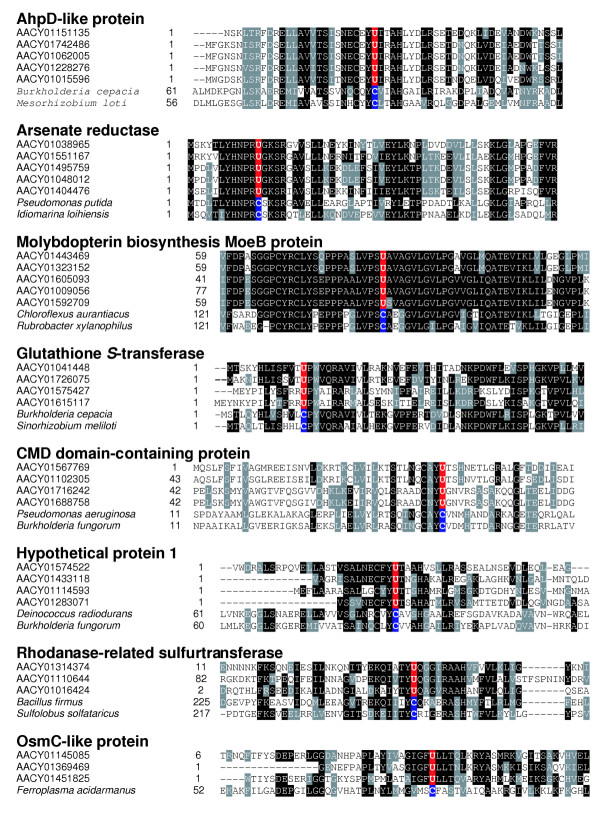
Multiple sequence alignments of new selenoproteins and their Cys homologs. The alignments show Sec-flanking regions in detected proteins. Both selenoprotein sequences detected in the Sargasso Sea database and their Cys-containing homologs present in indicated organisms are shown. Conserved residues are highlighted. Predicted Sec (U) and the corresponding Cys (C) residues in other homologs are shown in red and blue background, respectively. Sequence alignments were generated with ClustalW and shaded by BoxShade v3.21.

Among 15 new selenoprotein families, 13 contained at least two individual TGA-containing ORFs (Table 1). Although two selenoprotein families, DsbG-like and NADH:ubiquinone oxidoreductase, were represented by single entries, we placed them in the new selenoprotein category because they had been previously reported as candidate selenoproteins [22]. Of the 15 families, 14 either contained a domain of known function or were homologous to protein families with known functions, including several which were represented by multiple sequences: AhpD-like protein (27 sequences), arsenate reductase (14 sequences), molybdopterin biosynthesis MoeB protein (11 sequences), glutaredoxin (Grx) (ten sequences) and DsbA-like protein (nine sequences). Thus, these findings implicated selenium in arsenate reduction, molybdopterin biosynthesis, disulfide bond formation and other redox-based processes. No functional evidence could be obtained for one family, which was designated as hypothetical protein 1 (represented by four sequences). However, a conserved CXXU motif was present in hypothetical protein 1, suggesting a possible redox function. Multiple alignments of several new selenoproteins and their Cys-containing homologs (Figure 2) highlight sequence conservation of Sec/Cys pairs and their flanking regions.

**Figure 3:**
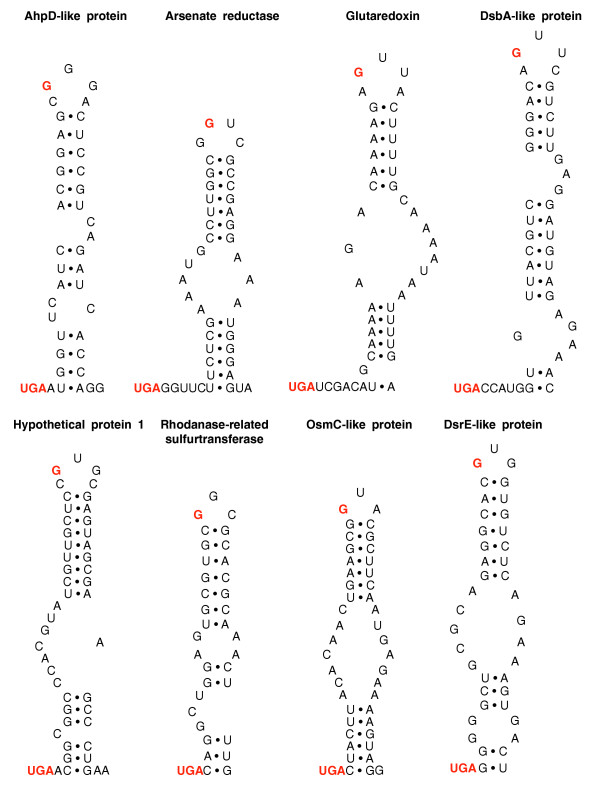
Predicted bacterial SECIS elements in representative sequences of some new selenoprotein families. Only sequences downstream of in-frame UGA codons are shown. In-frame UGA codons and conserved guanosines in the apical loop are shown in red. AhpD-like protein, AACY01418594; Arsenate reductase, AACY01238341; Glutaredoxin, AACY01002222; DsbA-like protein, AACY01178397; Hypothetical protein 1, AACY01574522; Rhodanase-related sulfurtransferase, AACY01016424; OsmC-like protein, AACY01145085; DsrE-like protein, AACY01486889.

**Figure 4:**
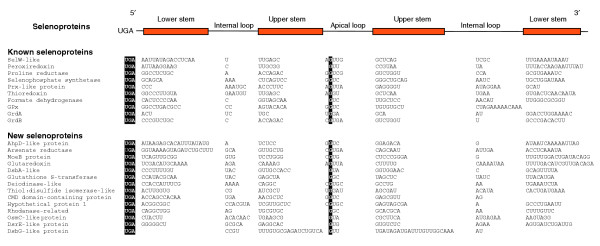
Alignment of SECIS elements present in Sargasso Sea selenoproteins. The Sargasso Sea dataset includes 10 known selenoprotein families and 15 new families. SECIS elements in representative members of these families were manually aligned on the basis of primary sequence and secondary structure features.

All new selenoproteins contained stable stem-loop structures downstream of Sec-encoding TGA codons that resembled bacterial SECIS elements. Representative predicted SECIS elements found in several new selenoprotein families are shown in Figure 3. A structural alignment of putative SECIS elements in known and new selenoprotein genes in the Sargasso Sea database (Figure 4) showed that they shared the common features of bacterial SECIS elements (for example, a small apical loop containing a guanosine, see Materials and methods).

### Significant overlap between eukaryotic and prokaryotic selenoproteomes

**Figure 5:**
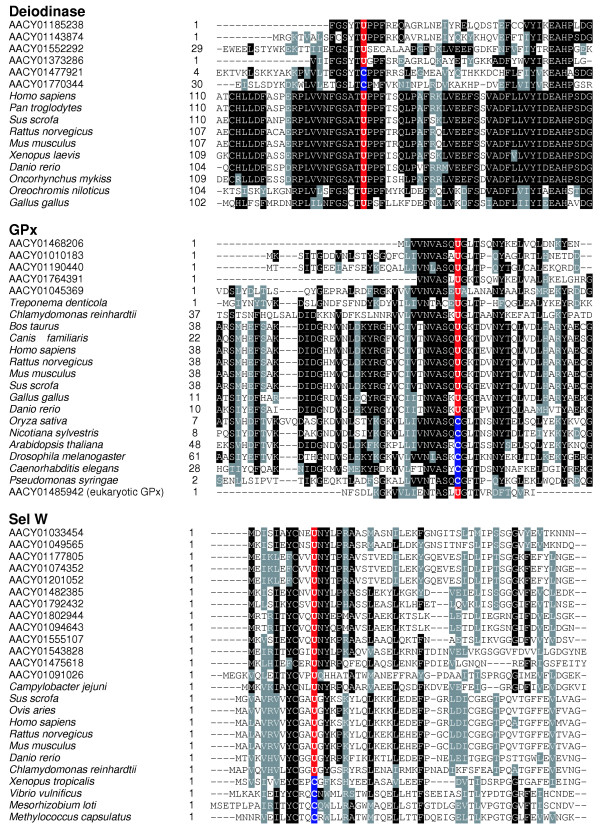
Multiple alignments of deiodinase, GPx and SelW. Conserved residues are highlighted. Predicted Sec (U) in selenoproteins and the corresponding Cys (C) residues in homologs are shown in red and blue background, respectively. Sequence alignments were generated with ClustalW and shaded by BoxShade v3.21.

**Figure 6:**
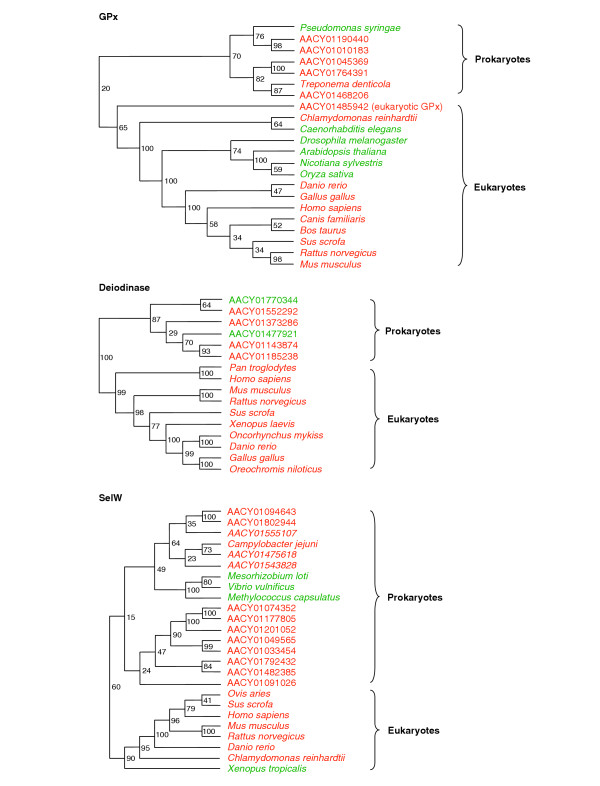
Phylogenetic analyses of deiodinase, GPx and SelW protein families. Selenoproteins are shown in red and Cys-containing homologs in green. The phylogenetic trees were generated by ClustalW and represented by Treeme.

**Figure 7:**
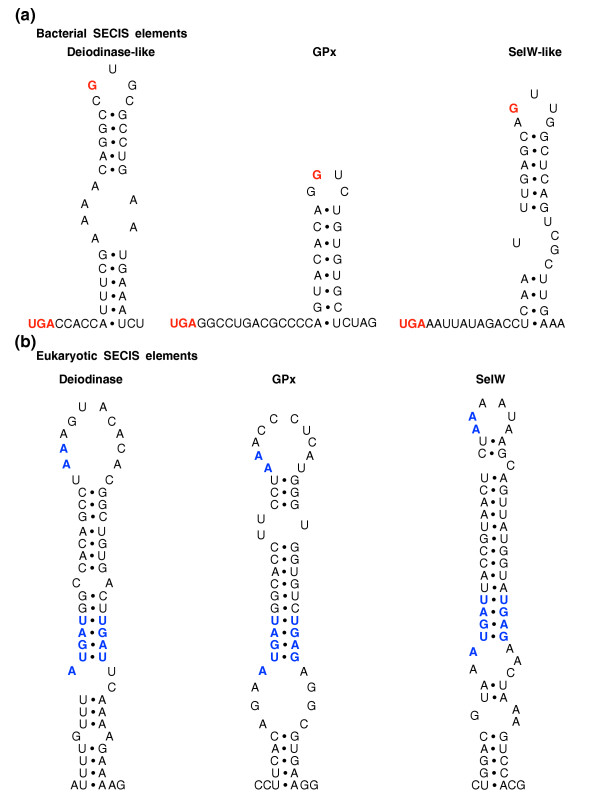
Comparison of bacterial and eukaryotic SECIS elements in deiodinase, GPx and SelW protein sequences. **(a) **In bacterial SECIS elements, only sequences downstream of in-frame UGA codons are shown and the conserved features (in-frame UGA codon and conserved G in the apical loop) are highlighted in red. **(b) **In eukaryotic SECIS elements, conserved features (quartet, AA in the apical loop or bulge and an A preceding the quartet) are shown in blue. Bacterial elements are from deiodinase-like protein, AACY01143874; GPx, AACY01190440; and SelW-like protein, AACY01475618. Eukaryotic elements are from deiodinase, NM_000792, *Homo sapiens*; GPx, X68314, *H. sapiens*; SelW, AY221261, *Danio rerio*.

Among 25 known and new bacterial selenoprotein families identified in the Sargasso Sea dataset, three families, SelW-like, GPx and deiodinase, were previously thought to be of eukaryotic origin. However, multiple sequence alignments (Figure 5) and phylogenetic analyses (Figure 6) strongly suggested a bacterial origin of these selenoproteins. Although several eukaryotic sequences in the Sargasso Sea dataset were also detected (for example, GPx homolog, accession number AACY01485942), all SelW and deiodinase sequences and most GPx sequences were bacterial selenoproteins. We based this conclusion on the presence of bacterial and absence of eukaryotic and archaeal SECIS elements in these sequences. In addition, phylogenetic analyses of coding sequences that flanked selenoprotein genes indicated that these contigs were derived from bacteria (data not shown). As information about the species present in the environmental samples is not available, analysis of SECIS elements provides a means of distinguishing selenoprotein sequences in the major domains of life, as SECIS elements are different in eukaryotes, bacteria and archaea in regard to sequence and structure [29]. Representative bacterial SECIS elements of the three bacterial selenoproteins and their eukaryotic counterparts are shown in Figure 7.

Deiodinase is known to activate or inactivate thyroid hormones via the reaction of reductive deiodination [30]. This protein has previously been described only in animals and only in the selenoprotein form. However, we identified both Cys-containing and Sec-containing homologs of deiodinase in the Sargasso Sea dataset (Figure 5). Bacterial deiodinase-like proteins likely serve a different function than animal deiodinases as thyroid hormones are not expected to occur in these organisms. Deiodinases possess a variation of the thioredoxin fold [31], which is known for redox functions. It is possible that bacterial deiodinase-like proteins also serve a redox function.

SelW and GPx homologs were recently detected in some bacteria, but the number of these sequences was small and their origin not clear [22]. Detection of a large number of SelW and GPx selenoprotein sequences in the Sargasso Sea allowed us to perform phylogenetic analyses (Figure 6), which suggested that at least some members of these families evolved independently in bacteria and eukaryotes.

In addition, we identified five eukaryotic selenoproteins: SelM, SelT, SelU, GPx and methionine-*S*-sulfoxide reductase (MsrA). Except for GPx, these families were represented by single selenoprotein genes. No bacterial SECIS elements were found in these genes. In SelM and SelT sequences, typical eukaryotic SECIS elements were present in 3' UTRs as detected by SECISearch [16], whereas GPx, MsrA and SelU sequences did not extend enough to test for presence of SECIS elements in 3' UTRs. However, the MsrA and GPx sequences were most similar to plant proteins, suggesting that the two proteins also were of eukaryotic origin. In addition, eukaryotic GPx sequences could be distinguished by the presence of introns.

Previous analyses of selenoprotein sets in the three domains of life revealed that bacterial and archaeal selenoproteomes significantly overlap, whereas eukaryotes had a different set of selenoproteins [15,20]. The only exception was selenophosphate synthetase, but as it is involved in Sec biosynthesis, this protein must be maintained in organisms that utilize Sec. However, our finding of additional selenoproteins in Sargasso Sea organisms revealed a significant overlap between prokaryotic and eukaryotic selenoproteomes.

### Differences in selenoprotein sets in the Sargasso Sea database and completely sequenced prokaryotic genomes

An exhaustive search of Sargasso Sea selenoproteins against 260 completely sequenced prokaryotic genomes revealed that these selenoproteins were present in a limited number of genomes, which contrasted with the widespread occurrence of their Cys-containing homologs (Table 2). Although the size of the Sargasso Sea dataset and the combined set of 260 prokaryotic genomes were similar, the two datasets differed in regard to both number and distribution of selenoprotein genes present in these databases. The Sargasso Sea dataset was three times richer in selenoproteins than the prokaryotic genomes, suggesting that the environment of the Sargasso Sea generally favors evolution and maintenance of selenoproteins. Presumably, the Sargasso Sea organisms take advantage of a relatively constant supply of selenium in sea water and have increased their demand for this trace element, whereas the dependence of the organisms with completely sequenced genomes on selenium is mixed as selenium may be a limiting factor in some environments. Six previously known selenoproteins were not detected in the Sargasso Sea database (Table 2). This is likely because these selenoproteins primarily occur in archaea. Archaea accounted only for a small fraction of the Sargasso Sea organisms [23].

In addition, the abundance of particular selenoprotein genes in the Sargasso Sea dataset and in the 260 microbial genomes was quite different. Particularly surprising was the small number of formate dehydrogenase genes in the Sargasso Sea database [32]. Previous analyses of completely sequenced prokaryotic genomes found that this protein was present in essentially all organisms that utilized Sec, and its occurrence was by far more common than any other selenoprotein [22]. However, in the Sargasso Sea environment, the utilization of this protein was limited. This might be related to the aerobic nature of microbial species that reside near the surface of the Sargasso Sea (where the environmental samples were collected for sequencing).

We also observed that in the previously analyzed prokaryotic genomes, more than half of selenoproteins were metal-binding proteins, in which Sec coordinated molybdenum, tungsten or nickel [22]. In contrast, the Sargasso Sea selenoproteins were primarily thiol-dependent peroxidases and oxidoreductases; metal-coordinating selenoproteins were represented exclusively by formate dehydrogenase and accounted for less than 4% of all detected selenoproteins. These data suggested that the previously characterized genomes did not represent the general composition of prokaryotic selenoproteomes.

Although the two sets of selenoproteins (Sargasso Sea and the completely sequenced prokaryotic genomes) were different, the majority of detected selenoproteins showed scattered occurrence. Indeed, the Sec-containing forms of proteins were rare compared to homologous Cys-containing forms, which were widespread. It appears that that most detected selenoproteins evolved recently from Cys-containing homologs in organisms, which already had the system for Sec insertion. It can be predicted that as searches of additional prokaryotic sequence datasets identify new selenoprotein genes, many of these will be present in only a small number of species. At present, Sec evolution is not fully understood, but it is clear that Sec/Cys interchanges are possible in both directions depending on the need for particular redox properties and on the restriction imposed by the dependence of species on the trace element selenium.

### Most selenoprotein families serve redox functions

Further analysis of both Sargasso Sea and completely sequenced prokaryotic genomes revealed that essentially all selenoproteins with known function were redox proteins, which used Sec either to coordinate redox-active metals or for thiol/disulfide-like redox catalysis. Among 25 selenoprotein families detected in the Sargasso Sea, 14 (194 selenoprotein sequences, 62.6%) were homologs of known thiol-dependent redox proteins (Table 3), and most other proteins were candidate redox proteins. Many of the Sargasso Sea selenoproteins contained a UXXC redox motif. The analogous CXXC motif is present in a variety of thiol-dependent redox enzymes [33-35], but it is also common in metal-binding proteins. The catalytic activity of UXXC-containing selenoenzymes is expected to be higher than that of its Cys-containing homologs [2,36]. In addition, several selenoproteins had other candidate redox motifs [34], such as UXXS (arsenate reductase), TXXU (peroxiredoxin and NADH:ubiquinone oxidoreductase), UXXT (glutathione peroxidase) and CXXU (AhpD-like protein [37], SelW-like protein, CMD domain-containing protein and hypothetical protein 1).

## Discussion

Whole-genome shotgun sequencing projects have been applied extensively to determine genomic sequences of a variety of organisms, and recently this approach was used to sequence the microbial community of the Sargasso Sea. Many of the Sargasso Sea organisms represent phyletic groups previously not known or poorly characterized, including organisms that could not be isolated from the microbial community or be cultured [23]. Identification of selenoprotein genes in such a large prokaryotic dataset may help understand the role of selenium in this microbial community and by analogy in other organisms, including humans.

Previous functional information on selenoproteins has been derived largely from wet-lab experiments. More recently, several *in silico *approaches that identify full sets of selenoproteins in organisms provided powerful new tools for determining identities of selenoproteins as well as their expression characteristics and functions [16-20,38]. Most of these methods were based on searches for SECIS elements. As Sec is typically located at enzyme active centers, and most selenoproteins had homologs in which Cys replaced Sec, a SECIS-independent strategy was also developed that allowed searches for Sec/Cys pairs in homologous sequences [21,22].

In the present study, we used a similar procedure, but supplemented it with additional filters to improve performance. All known prokaryotic selenoprotein families present in the Sargasso Sea genomic dataset were identified by this approach (209 genes that clustered into ten prokaryotic selenoprotein families). In addition, 101 sequences that belonged to 15 new selenoprotein families were identified. Thus, our study has approximately doubled the list of known prokaryotic selenoprotein families and generated the largest selenoprotein dataset to date.

On the basis of the presence of SECIS elements specific to major domains of life, we could determine the origin of detected selenoproteins (that is, bacterial, archaeal or eukaryotic). All ten known and 15 new prokaryotic selenoprotein families had predicted bacterial SECIS elements. Interestingly, both selenoprotein forms and Cys-containing homologs of thyroid hormone deiodinase, a protein previously thought to be restricted to the animal kingdom and present exclusively in the selenoprotein form, were identified in prokaryotes. The detected deiodinase-like proteins were prokaryotic as they contained bacterial SECIS elements.

Detection of prokaryotic deiodinase-like proteins and several other bacterial selenoproteins thought to be restricted to eukaryotes suggests a revision of the view that eukaryotic and prokaryotic selenoproteomes do not overlap. Although this idea was consistent with the previous selenoprotein analyses, at least four selenoprotein families are now known that occur in both prokaryotes and eukaryotes: SelW, GPx, selenophosphate synthetase and deiodinase. We also detected homologs of five additional eukaryotic selenoproteins, but the absence of bacterial SECIS elements, presence of eukaryotic SECIS elements or introns, and homology to eukaryotic proteins argued that these selenoproteins were eukaryotic in origin.

Surprisingly, sets of selenoproteins in the Sargasso Sea database and in the combined set of 260 completely sequenced prokaryotic genomes were quite different in regard to both identities and number of selenoprotein genes. The Sargasso Sea dataset was rich in selenoprotein genes, most of which were homologs of known thiol-dependent redox enzymes. In contrast, the proportion of selenoprotein genes in completely sequenced prokaryotic genomes was approximately three times lower, and the majority of detected genes used Sec for metal coordination. Thus, even with the availability of 260 genomes, the roles of selenium in nature are just beginning to be understood. For example, our current analysis of the Sargasso Sea dataset implicated selenium in arsenate reduction, molybdopterin biosynthesis, sulfurtransferase function and other processes, which were not known to be dependent on this trace element.

We also observed common features in the two sets of selenoproteins. For example, most of the detected selenoproteins had a large number of Cys homologs. The scattered occurrence of selenoproteins in both datasets suggests a highly dynamic nature of Sec evolution. As long as the system for Sec insertion is maintained, Sec may appear when required by the changing environment and disappear when this requirement recedes. Thus, the analysis of selenoproteomes and the compensatory sets of Cys-containing proteins provides a unique model system to examine evolutionary forces to a changing environment.

## Materials and methods

### Sequence databases and resources

The whole-genome shotgun sequence database of the Sargasso Sea was obtained from the National Center for Biotechnology Information (NCBI) ftp server with the project accession number AACY00000000 [39]. Unlike conventional sequence entries, only the unassembled singletons and individual singletons were deposited in order to accurately reflect the diversity in the sample and to allow searches across the entire sample within a single database. The Sargasso Sea database contained 811,372 genomic sequences, which corresponded to a total of 1.045 billion nucleotides.

The NR protein database was downloaded from the NCBI ftp server [40]. This dataset contained 1,990,024 protein sequences (667,623,348 amino acids). Blast programs [41] were also obtained from the NCBI ftp server [42]. We used the 2.2.9 version of this program.

To enable selenoprotein searches automatically, we developed a set of programs as discussed below. A UNIX/LINUX platform was used. The entire search process was performed on a Prairiefire 128-node, 256-processor Beowulf cluster supercomputer at the Research Computing Facility of the University of Nebraska - Lincoln.

### Identification of Cys/TGA pairs in homologous sequences

Each Cys-containing sequence in the NR protein database was searched against the Sargasso Sea database of nucleotide sequences for possible TGA-containing hits using TBLASTN. E-value cutoff was set to 10.0. TBLASTN output for each protein sequence was parsed and Cys/TAA or Cys/TAG pairs were filtered out. Only local alignments, in which Cys in a query sequence was aligned with TGA in the nucleotide sequence from the target Sargasso Sea database, were further analyzed. As Sec is typically located in enzyme active sites, additional filters were added. Specifically, local alignments were discarded if they contained more than two stop codons (including TGA, TAA and TAG), two stop codons of which one was not TGA, or two TGA codons with one aligned to a non-Cys residue. A total of 38,446 local redundant alignments (also designated as Cys/TGA pairs) were identified which corresponded to 19,410 proteins in the NR protein database.

### Analyses of ORFs, conservation of TGA-flanking regions and redundancy

For each TGA-containing sequence in the local alignment set, regions upstream and downstream of the TGA were analyzed to identify minimal ORFs with the assumption that in-frame TGA coding for Sec must be inside predicted ORFs. If stop codons were encountered closer to TGA codons than candidate start codons (ATG or GTG), such TGA-containing sequences were discarded. Conservation of TGA-flanking regions in all 6 reading frames was also analyzed with BLASTX and screened against a database of conserved domains using RPS-BLAST. These criteria were also used to filter out false positive hits. Finally, redundant sequences were removed. These filters reduced the set to 2,131 unique TGA-containing candidate ORFs.

### Clustering of TGA-containing sequences

To cluster protein sequences into different protein families or groups, the pairwise alignment tool in the BLAST program package, BL2SEQ, was used. 1,045 clusters were obtained with 1 to 63 sequences in each cluster.

### Cysteine conservation and selenoprotein classification

Considering that Cys/TGA pairs in most false-positive hits were not expected to be conserved, whereas conservation was expected for true-positive Cys/Sec pairs, all clusters were automatically searched against NCBI NR and microbial databases using BLASTX and TBLASTX. Each predicted ORF containing an in-frame TGA was considered further only if at least two corresponding Cys-containing homologs were detected and the proportion of TGA/Cys pairs in the set of homologs was greater than 50%. This procedure resulted in 331 clusters containing 1,072 ORFs.

All 331 clusters were analyzed for the presence of potential bacterial SECIS elements immediately downstream of the TGA codons using mfold [43] or RNAfold [44] programs. In addition, candidate SECIS structures were screened against a bacterial SECIS consensus model [45]. The presence of archaeal or eukaryotic SECIS elements was tested using SECISearch [20,22]. The occurrence of SECIS elements specific for each domain of life was one criterion to determine protein origin. Phylogenetic analyses and the occurrence of introns were also used as criteria for designating proteins as bacterial, archaeal or eukaryotic.

A simple classifier was developed to divide clusters that contained bacterial SECIS-like structures into three groups: known selenoproteins, new selenoproteins and selenoprotein candidates. Except for known selenoproteins, clusters containing at least two different sequences with conserved in-frame TGA codons were considered as new selenoproteins. Clusters containing only one sequence were considered selenoprotein candidates because of the possibility of a sequencing error causing an in-frame TGA. Finally, clusters that could be aligned such that their TGAs also aligned, were joined into larger clusters.

## Additional data files

The complete set of predicted selenoprotein sequences with annotations (accession number, protein name, ORF location and in-frame TGA location) is available as a text file (Additional data file 1) with the online version of this paper and at [46].

## Supplementary Material

Additional File 1 A complete set of predicted selenoprotein sequences with annotations (accession number, protein name, ORF location and in-frame TGA location) Click here for file

## Figures and Tables

**Table 1:** Selenoprotein families identified in the Sargasso Sea database

Prokaryotic selenoprotein family	Unique sequences	COG/Pfam ID	COG/Pfam description
Known selenoproteins (209 sequences)			
SelW-like protein	48	Pfam05169	Selenoprotein W-related
Peroxiredoxin (Prx)	43	COG1225	Peroxiredoxin
Proline reductase (PrdB)	42	-	
Selenophosphate synthetase	28	COG0709	Selenophosphate synthetase
Prx-like protein	22	COG0450	Peroxiredoxin-like
Thioredoxin (Trx)	11	COG3118	Thioredoxin
Formate dehydrogenase alpha chain (fdhA)	8	COG0243	Anaerobic dehydrogenases
Glutathione peroxidase (GPx)	5	COG0386	Glutathione peroxidase
Glycine reductase selenoprotein A (grdA)	1	-	
Glycine reductase selenoprotein B (grdB)	1	Pfam07355	Glycine reductase selenoprotein B
			
New selenoproteins (101 sequences)			
AhpD-like protein	27	COG2128	Uncharacterized conserved protein
Arsenate reductase	14	COG1393	Arsenate reductase and related proteins
Molybdopterin biosynthesis MoeB protein	11	COG0476	Dinucleotide-utilizing enzymes, molybdopterin biosynthesis
Glutaredoxin (Grx)	10	COG0695	Glutaredoxin and related proteins
DsbA-like protein	9	COG2761	DsbA-like
Glutathione S-transferase	4	COG0625	Glutathione S-transferase
Deiodinase-like protein	4	Pfam00837	Iodothyronine deiodinase
Thiol-disulfide isomerase-like protein	4	-	
CMD domain-containing protein	4	Pfam02627	Carboxymuconolactone decarboxylase
Hypothetical protein 1	4	-	
Rhodanese-related sulfurtransferase	3	COG2897	Rhodanese-related sulfurtransferase
OsmC-like protein	3	COG1765	Predicted redox protein, OsmC-like
DsrE-like protein	2	Pfam02635	DsrE-like
DsbG-like protein	1	COG1651	DsbG, Protein-disulfide isomerase
NADH:ubiquinone oxidoreductase	1	COG2209	NADH:ubiquinone oxidoreductase
Total	310		

Classification of selenoproteins (10 previously known and 15 new prokaryotic selenoprotein families) is supported by COG or Pfam sequence clusters (or both) as shown in this table. The number of individual selenoprotein sequences for each family is indicated.

**Table 2:** Comparison of selenoproteins identified in the Sargasso Sea database and in the combined set of completely sequenced prokaryotic genomes

Prokaryotic selenoprotein family	Sequences in the Sargasso Sea database		Sequences in completely sequenced prokaryotic genomes	
		
	Selenoprotein	Cys homolog	Selenoprotein	Cys homolog
Known selenoproteins detected in the Sargasso Sea dataset				
SelW-like protein	48	7	4	20
Peroxiredoxin	43	Widespread*	1	Widespread
Proline reductase PrdB	42	1	1	5
Selenophosphate synthetase	28	23	16	22
Prx-like protein	22	4	2	6
Thioredoxin	11	Widespread	2	Widespread
Formate dehydrogenase alpha chain	8	Widespread	40	Widespread
Glutathione peroxidase	5	Widespread	1	Widespread
Glycine reductase selenoprotein A	1	0	5	0
Glycine reductase selenoprotein B	1	1	5	2
				
New selenoproteins detected in the Sargasso Sea dataset				
AhpD-like protein	27	Widespread	0	Widespread
Arsenate reductase	14	Widespread	0	Widespread
Molybdopterin biosynthesis MoeB protein	11	Widespread	0	Widespread
Glutaredoxin	10	17	1	Widespread
DsbA-like protein	9	Widespread	0	Widespread
Glutathione S-transferase	4	Widespread	0	Widespread
Deiodinase-like protein	4	6	0	0
Thiol-disulfide isomerase-like protein	4	Widespread	0	Widespread
CMD domain-containing protein	4	14	0	5
Hypothetical protein 1	4	7	0	5
Rhodanase-related sulfurtransferase	3	Widespread	0	Widespread
OsmC-like protein	3	10	0	17
DsrE-like protein	2	3	1	9
DsbG-like protein	1	Widespread	1	Widespread
NADH:ubiquinone oxidoreductase	1	Widespread	0	Widespread
				
Known selenoproteins not detected in the Sargasso Sea dataset				
Formylmethanofuran dehydrogenase	0	Widespread	4	Widespread
F420-reducing hydrogenase alpha subunit	0	4	4	Widespread
F420-reducing hydrogenase, delta subunit	0	1	3	Widespread
Methylviologen-reducing hydrogenase	0	3	3	Widespread
Heterodisulfide reductase	0	0	4	23
HesB-like protein	0	2	7	2
Total	310		105	

*Widespread, occurrence in more than 40 sequences in the Sargasso Sea database or more than 40 in organisms in the combined set of completely sequenced prokaryotic genomes.

**Table 3:** Thiol-dependent redox selenoproteins detected in the Sargasso Sea database and their predicted redox motifs containing Sec (U)

Selenoprotein family	Redox motif	Individual sequences
Known redox proteins		
Peroxiredoxin	TXXU	43
Proline reductase PrdB	UXXC	42
Prx-like protein	UXXC	21
	UXC	1
Thioredoxin	UXXC	11
Glutathione peroxidase	UXXT	5
AhpD-like protein	CXXU	27
Arsenate reductase	UXXS	14
Glutaredoxin	UXXC	10
DsbA-like protein	UXXC	9
Thiol-disulfide isomerase-like protein	UXXC	4
OsmC-like protein	UXXT	3
DsrE-like protein	UXXC	2
DsbG-like protein	UXXC	1
NADH:ubiquinone oxidoreductase	TXXU	1
		
Candidate redox proteins		
SelW-like protein	CXXU	48
CMD domain-containing protein	CXXU	4
Hypothetical protein 1	CXXU	4
